# Tumoral Prostate Shows Different Expression Pattern of Somatostatin Receptor 2 (SSTR2) and Phosphotyrosine Phosphatase SHP-1 (PTPN6) According to Tumor Progression

**DOI:** 10.1155/2009/723831

**Published:** 2009-04-12

**Authors:** Ariel Ernesto Cariaga-Martinez, María Angelica Lorenzati, Mario Alejandro Riera, Marisa Angelica Cubilla, Andrés De La Rossa, Ernesto Martín Giorgio, María Mercedes Tiscornia, Esteban Mariano Gimenez, María Eugenia Rojas, Bárbara Julieta Chaneton, Dora Isabel Rodríguez, Pedro Darío Zapata

**Affiliations:** Laboratorio de Biologia Molecular, Facultad de Ciencias Exactas Aplicada Químicas y Naturales, Universidad Nacional de Misiones, N3300LQH Posadas, Argentina

## Abstract

Prostate proliferation is dependent of androgens and many peptide hormones. Recent reports suggest that SSTR2 and SHP-1 were two fundamental components on antiproliferative effect of somatostatin. Many studies on SHP-1 revealed that the expression of this protein was diminished or abolished in several of the cancer cell lines and tissues examined. However, it is necessary to confront the cell lines data with real situation in cancer cases. Our studies have shown that epithelial expressions of both proteins, SHP-1 and SSTR2, in normal and benign hyperplasia are localized in the luminal side of duct and acinar cells. Also, SSTR2 is expressed in stromal cells. In malignant prostate tissue, SHP-1 was diminished in 28/45 cases or absent in 12/45 cases, whereas SSTR2 epithelial was diminished in 38/45 cases or lost in only 2/45 cases. The intensity of immunostained was highly negative correlated with Gleason grade for two proteins.

## 1. Introduction

Prostate cancer is one of the most common malignancies among men in the Western world and is a great health problem in many countries. As the tumor is initially androgen dependent in the majority of cases, endocrine manipulation is a first-line therapy for metastatic and locally advanced cancer and often achieves remission or stabilization of the disease [1]. However, this remission period is invariably followed by tumor relapse, and the available treatment options, based on cytotoxic chemotherapy or aromatase inhibitors, are only palliative. Finally, patients with metastasic prostate cancer develop an androgen refractory phenotype that leads to disease progression and eventual death [2].

The prostate is not exclusively dependent on androgens, but also on additional factors of paramount importance for maintaining normal prostatic function that play a role in pathological conditions development. In this sense, the importance of peptide hormones, growth factors, autocrine-paracrine regulatory loops, and stromal-epithelial interactions is now widely recognized [3].

Protein tyrosine phosphorylation is regulated in the cell by the opposing activities of two enzymes: protein tyrosine kinases (PTKs), which transfer phosphate from ATP to substrate proteins, and protein tyrosine phosphatases (PTPs), which remove it. Functionally, the most important effect of tyrosine phosphorylation is to create high-affinity binding sites for other proteins containing small modular phosphotyrosine- (pTyr-) binding domains, most notably Src homology 2 (SH2) domains [4]. Any deviation in balance between PTP and PTK can promote abnormal cell proliferation and differentiation thereby resulting in different kinds of diseases [5–7]. SHP-1 (PTPN6) is one of the most important component from this equilibrium and decreased activity, gene mutation or gene deletion, leading to an increase in tyrosine-phosphorylated proteins in cells with pathological consequences [8–11]. 

Somatostatin (SST) was described initially as a secretory product of the hypothalamus acting as a potent inhibitor of GH secretion [12]. Subsequently, high densities of SST-producing neuroendocrine cells have been localized throughout the central and peripheral nervous systems, in the endocrine pancreas and in the gut, and to a lower extent in the thyroid glands, adrenals glands, submandibular glands, kidneys, liver, colon, rectum, small intestine, stomach, placenta, and prostate [13, 14]. SST is classically known to inhibit the secretion of a wide range of hormones, exocrine glands, and gastrointestinal motility. Among other findings is the inhibition of immunoglobulin synthesis and lymphocyte proliferation in lymphoid tissues [15]. Last but not least, SST has revealed an antiproliferative potential, reversing the impact of mitogenic signals delivered by substances such as epidermal growth factor (EGF) and somatomedin C/insulin-like growth factor-1 (IGF-1) (reviewed in [16]). These actions are mediated by a family of seven transmembrane (TM) domain G-protein-coupled receptors that comprise five distinct subtypes (termed SSTR1 to 5). SSTRs are widely expressed in many tissues, frequently as multiple subtypes that coexist in the same cell [14]. The five receptors share common signaling pathways such as the inhibition of adenylate cyclase, activation of phosphotyrosine phosphatase (PTP), and modulation of mitogen-activated protein kinase (MAPK) through G-protein-dependent mechanisms. Among somatostatin receptors, SSTR2 has been found to play a critical role in the negative control of cell growth and to act as a tumor suppressor gene for pancreatic cancer [17] and in medullary thyroid carcinoma [18]. Many reports suggest that SHP-1 may participate in the negative regulation of cellular proliferation by SST [19, 20] and other peptides like AT-II [21–24]. Interestingly, the presence of SHP-1 (PTPN6) was identified in rat prostate [25] and in human prostate [20].

Extensive studies on SHP-1 protein and mRNA revealed that the expression of SHP-1 protein was diminished or abolished in most of the cancer cell lines and tissues examined (reviewed in [26]). Similarly, growth of cancer cells was suppressed after introducing the SHP-1 gene into the corresponding cell lines [27, 28]. These data suggest that SHP-1 can play either negative or positive roles in signal transduction pathways regulation [29, 30]. Dysfunction in SHP-1 regulation can cause abnormal cell growth and induce different kinds of cancers. These findings support the hypothesis that the SHP-1 gene functions as a tumor suppressor.

On the other hand, some studies demonstrate variations on SSTR2 distribution on tumor and normal specimens and these associations with histological grade, proliferation, and treatment of tumors [31–34].

The aim of this study is evaluate the expression of both SSTR2 and SHP-1 (PTPN6) in normal prostate and their variation through tumoral progression in human prostate cancer (different Gleason scores). 

## 2. Materials and Methods

### 2.1. Tissue Samples

Human prostate tissues were obtained from transrectal needle biopsies, transurethral resection, and retropubic or radical prostatectomy, from patients attend at “Dr. Ramón Madariaga” Public Hospital Pathology Service from Posadas, Misiones. The tissues were used in the experiments after approval by the local ethical committee. 

Tissues were fixed in formalin and embedded in paraffin blocks. Sections were cut at 4 *μ*m and stained with hematoxylin-eosin for histological diagnosis, with additional sections cut for immunostaining. 

Specimens were graded according to the original report Gleason grading and classified with specimens with high-grade adenocarcinomas zones represented by Gleason grade 4 or 5 (*n* = 20); specimens with intermediate-grade adenocarcinomas zones represented by Gleason grade 3 (*n* = 13); specimens with low-grade adenocarcinomas zones represented by Gleason grade 1 or 2 (*n* = 12). 

### 2.2. Immunohistochemistry

The antibodies against for SHP-1 (sc-287, Santa Cruz Biotechnology, Inc) and SSTR2 (sc-25676, Santa Cruz Biotechnology, Inc) were used. The immunostaining was performed by using the Universal DakoCytomation Labelled Streptavidin-Biotin2 System, Horseradish Peroxidase (LSAB2 System - HRP, DAKO), and according to manufacturer's protocol. Specimens of hyperplasic prostate and leukocytes, known to express SHP-1 were used as positive controls. Primary antibody was replaced with buffer as a negative control of immunostained tissues.

After deparaffinization and rehydration sections were treated with 3% hydrogen peroxide for 5 minutes. After that, sections were incubated with primary antibody (Dilution 1:100) and left overnight in moist chambers at 4°C followed by sequential 30-minute incubation with a biotinylated link antibody and 10-minute incubation peroxidase-labelled streptavidin. After every antibody incubation, slides were washed 3 times in TTBS buffer for 5 minutes. Staining is completed after 5-minute incubation with 3-3′ diaminobenzidine (DAB) Substrate Chromogen generating a brown-colored precipitate at the antigen site. The sections were then rinsed in distilled water for 5 minutes and counterstained with 50% Mayer hematoxylin solution. After dehydration, slides were mounted with Canada balsam (Merck, Darmstadt, Germany). Slides were analyzed under light microscopy and photographed.

### 2.3. Analysis of Immunostained Tissues

Gleason classification was established, and the immunostaining was compared with prostate hyperplasia (positive control). Morphological assessment of immunostained tissue was performed with the aid of an occular grid, photographed and analyzed. In cases showing zones with different tumoral grade, the immunostaining of each zone was analyzed.

Before slides observation, 3 representative photographies of every tumoral grade in each case were analyzed. The immunostaining intensity was classified as “+++” if the intensity was the same of positive control, lightly reduced (“++”) or low (“+”) if the intensity was less than positive control, and negative in absence of immunoreactivity.

### 2.4. Statistic Analysis

The immunostaining and Gleason grade were codified with numeric code. Correlation between staining intensity and tumor grade for two proteins was evaluated by Pearson Correlation Coefficient (*r*), that quantifies the direction and magnitude of correlation, using GraphPad Prism version 4.00 for Windows, GraphPad Software, San Diego, Calif, USA, http://www.graphpad.com.

## 3. Results

**Figure 1:**
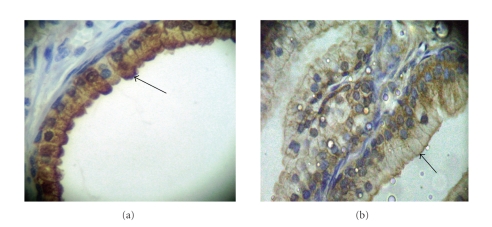
Immunohistochemical pattern of SHP-1 and SSTR2 on hyperplasic human prostate. The picture has shown the characteristic SHP-1 (a) and SSTR2 (b) immunostaining on benign prostate hyperplasia hyperplasic. The intensity was classified as “+++” for comparison with tumor glands.

In the normal prostate tissues and hyperplasic prostate of positive controls, the majority of epithelial cells showed granular cytoplasmatic immunostaining for SHP-1 and SSTR2 restricted to the luminal side of duct and acinar cells (Figure 1). In this tissues, the immunostainings intensity was classified as “+++” for the both of proteins. However, the stromal shown expression only for SSTR2 with low intensity classified as “+” in comparison with epithelial cells.

Most of cancer biopsies showed different tumor grade zones; SHP-1 and SSTR2 immunostaining were analyzed for each zone and showed different intensities in comparisons with the control. 

**Figure 2:**
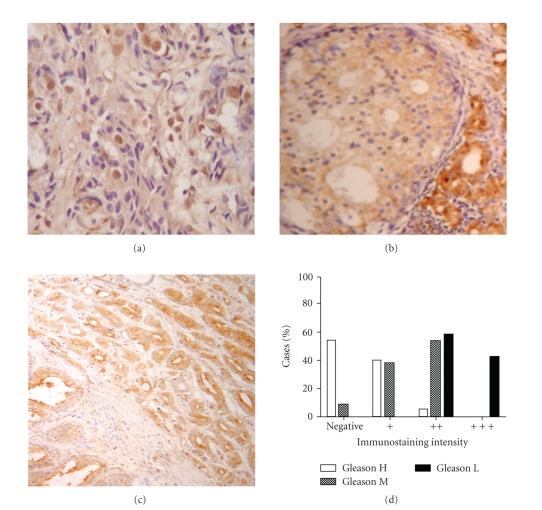
Distribution of SHP-1 on human prostate carcinoma. Photographies show characteristic pattern of SHP-1 immunostaining on tumor zones: (a) Gleason H; (b) Gleason M; (c) Gleason L. (d) Percentages of cases classified for immunostaining intensity and Gleason grade. Tissues were immunostained for SHP-1 was and the immunostaining intensity was classified as “+++” if the intensity was the same of positive control, “++” or “+” if the intensity was less than positive control, and “−” if absent. Gleason L: low-grade adenocarcinomas zones, Gleason grade 1 and 2. Gleason I: intermediate-grade adenocarcinomas zones, Gleason grade 3. Gleason H: high-grade adenocarcinomas zone, Gleason grade 4 and 5.

The patterns of immunoreactivity of SHP-1 were normal in only 5/45 cases (11%), diminished (“+” or “++”) in 28/45 cases (62%), and lost in 12/45 cases (27%) in agreement to increase of Gleason grade with Pearson Correlation Coefficient of −0.8061 (Figure 2). This value indicates that SHP-1 immunostain decreases as Gleason grade increases. 

Frequently, high-grade adenocarcinomas zones, represented by Gleason grade 4 or 5, were negative in 11/20 cases or showed low SHP-1 expression (“+”) in 8/20 cases. Intermediate-grade adenocarcinomas zones, represented by Gleason grade 3, were negative only in 1/13 case, showed low SHP-1 expression (“+”) in 5/13 cases or lightly reduced expression (“++”) in 7/13 cases. However, low-grade adenocarcinomas zones, represented by Gleason grade 1 or 2, only showed lightly reduced SHP-1 expression (“++”) in 7/12 cases. 

**Figure 3:**
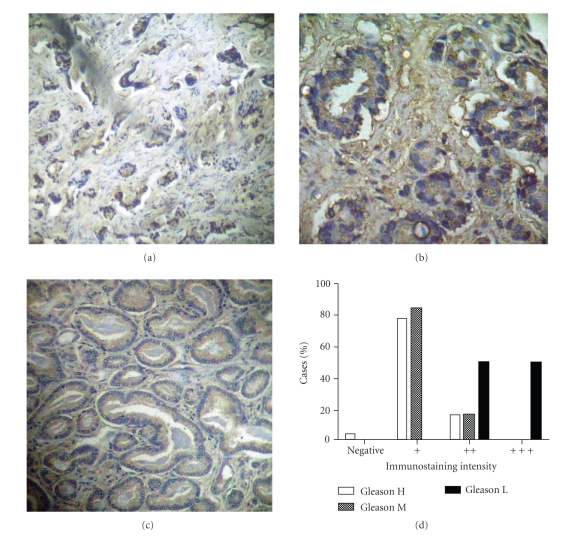
Distribution of SSTR2 on human prostate carcinoma. Photographies show characteristic pattern of SSTR2 immunostaining on tumor zones: (a) Gleason H; (b) Gleason M; (c) Gleason L. (d) Percentages of cases classified for immunostaining intensity and Gleason grade. Tissues were immunostained for SSTR2 and the immunostaining intensity was classified as “+++” if the intensity was the same of positive control, “++” or “+” if the intensity was less than positive control, and “−” if are absent. Gleason L: low-grade adenocarcinomas zones, Gleason grade 1 and 2. Gleason I: intermediate-grade adenocarcinomas zones, Gleason grade 3. Gleason H: high-grade adenocarcinomas zone, Gleason grade 4 and 5.

For SSTR2 the immunostained patterns were normal in only 5/45 cases (11%), diminished (“+” or “++”) in 38/45 cases (84%), and lost in only 2/45 cases (4%) in agreement to increase of Gleason grade with Pearson Correlation Coefficient of −0.7245 (Figure 3). This value indicates that SSTR2 immunostain decreases as Gleason grade increases.

High-grade adenocarcinomas zones, represented by Gleason grade 4 or 5, were negative in 1/20 case, showed low SSTR2 expression (“+”) in 15/20 cases or lightly reduced expression (“++”) in 4 cases. Intermediate-grade adenocarcinomas zones, represented by Gleason grade 3, showed low SSTR2 expression (“+”) in 11/13 cases or lightly reduced expression (“++”) in 2/13 cases. However, low-grade adenocarcinomas zones, represented by Gleason grade 1 or 2, showed lightly reduced SSTR2 expression (“++”) in 6/12 cases or were normal. 

## 4. Discussion

The underlying mechanisms for tumoral prostate growth are still poorly understood. It is now clear that PTPs play an important role exerting negative or positive effects in cancer-related signaling pathways. Deviation from this equilibrium can be induced by decreasing PTPs activity or expression resulting in gene mutation or gene transcription downregulation [4, 28]. SHP-1 (PTPN6) and SHP-2 (PTPN11) are key regulators that control the intracellular phosphotyrosine level in lymphocytes and epithelial cells [7, 8, 10, 18, 20, 23, 27, 32, 35]. Some works demonstrated the antiproliferative effect of somatostatin and other peptides in human prostate gland. For these peptides, the evidence has indicated that protein tyrosine phosphatase SHP-1 plays a crucial role in signal transduction mechanisms [26, 28]. Numerous studies reported that all somatostatin receptor subtypes are able to stimulate SHP-1 as the PTP involved in the somatostatin-induced antiproliferative signal [13, 18–20, 23, 24, 26, 27]. In fact, previous studies have revealed that SHP-1 becomes activated in response to somatostatin and SSTR2 association and participates in negative regulation of mitogenic insulin signaling [23, 24, 27].

Human prostate biopsies and studies of SHP-1 or SSTR2 expression in prostate carcinoma are limited (reviewed in [26]). Our studies have shown the epithelial expressions of both proteins, SHP-1 and SSTR2, in normal and benign hyperplasia are localized in the luminal side of duct and acinar cells. In addition, SSTR2 is expressed in stromal cells, in agreement with previous studies [34, 36–40].

In malignant prostate tissue, our results showed that SHP-1 immunoreactivity was diminished in 62% or was absent in 26% of cases, whereas SSTR2 epithelial immunostaining was diminished in 84% or was absent in 3% of cases. In addition, the intensity of immunostained and the patterns of immunoreactive tumor cells were highly negative correlated with Gleason grade for two proteins, indicating that SHP-1 immunostain decreases than SSTR2 as Gleason grade increases; that is the protein expression is lower in high Gleason grade.

Previous studies had shown stronger distributions of transcripts for SSTR2 in cancer prostate without correlation between mRNA of SSTR2 and Gleason grade in appearance contradiction with our results, which shows a diminished protein expression with an increase in tumor Gleason grade [36, 39]. These observations should be due to at mRNA variations not is direct correlated with protein variations and is probably that the protein expression is controlled at the posttranscriptional level.

All observations have indicated that SSTR2 expression and distribution in cancer prostate epithelium cells are stronger and could cause an abnormal response of tumor at somatostatin analogs treatment. For this, we think that SSTR2 screening is necessary after incorporate therapy with any somatostatin agonists like AN-238 [41–43].

Many studies suggest the important role of SHP-1 in cell proliferation and analyzed the SHP-1 expression and its contribution to the development of a malignant phenotype. In lymphocytes, SHP-1 binds the immunoreceptor tyrosine-based inhibition motif (ITIM) of the inhibitory receptors (CD22, CD72, Fc*γ*RIIB, p70_NKB1, KIR) through its SH2 domains and subsequently activates or deactivates signals regulating other pathways through tyrosine phosphatase activity [26]. Dysfunction of SHP-1 induces lymphoma, leukemia, and other related diseases. Delibrias et al. reported the SHP-1 activity and expression diminished in many EBV-positive Burkitt's lymphomas cell lines [44]. Similarly, the pattern of SHP-1 expression is greatly diminished or suppressed in T lymphoma cell lines [35, 45, 46]. Decreased or suppressed SHP-1 expression in both malignant transformation and tumor cell invasiveness in most lymphoma/leukemia cell lines and in specimens from related cancer patients is similar, thus supporting the crucial role of SHP-1 in the pathogenesis in lymphomas [47]. 

However, established cell lines may not truly represent the in vivo situation, and it is necessary to confront this data with situations in biopsies of cancer cases. To determine that the SHP-1 expression patterns in lymphoma/leukemia specimens were essentially consistent to those observed in the corresponding cell lines, Oka et al. analyzed 207 paraffin-embedded specimens of various malignant lymphomas/leukemia using cDNA expression array and tissue microarray techniques. These results revealed that 100% of NK/T cell lymphoma specimens and more than 95% of various types of malignant lymphoma specimens (DLL, FL, HD, MCL, PT and ATLL) were negative for SHP-1 protein expression [35].

Also, SHP-1 can activate an antiproliferation signaling pathway in breast cancer cells [48], pancreatic cancer [19], and prostate cancer [20]. In prostate carcinoma, the androgen deprivation can also lead to development of a negative growth-regulating loop involving antiproliferative peptides like somatostatin. Previous studies revealed that somatostatin is produced by human prostate cells lines PC-3 and LNCaP, and this peptide is an important negative regulator of the proliferation of these cell lines. These cells have shown somatostatin receptor 2 (SSTR2) and somatostatin receptor 5 (SSTR5) expressions [20, 28]. Zapata et al. clearly demonstrate that SHP-1 is a component involved in the somatostatin autocrine inhibitory loop: when somatostatin secreted was blocked, the PC3 cell proliferation increased and SHP-1 activity decreased; when somatostatin was overexpressed, the PC3 cell proliferation decreased and SHP-1 activity levels increased [20]. On the other hand, Valencia et al. indicated that PC3 cell line expresses other PTPs, such as SHP-2 and PTP1B, which could also mediate the antiproliferative effect of somatostatin in the prostate [25]. However, this loop could be deficient due to low expression of SHP-1. Regardless the levels of peptides receptors on prostate cancer (affected or not by tumor progression) [49], postreceptor signaling defects, such as loss of SHP-1, may play a role in the pathogenesis of prostate cancer by short-circuiting in signaling pathways of these antiproliferative peptides with persistence of signals generated by growth factors. In this sense, Douziech et al. have demonstrated that SHP-1 expression conditioned the somatostatin effects over human pancreatic cancer growth, the peptide antiproliferative effects were not observed when the enzyme was not expressed [23]. Here we observed a diminished or lost expression of SHP-1 and SSTR2, the two fundamental components of antiproliferative signal presents in prostate cells, and we demonstrate that this is consistent with advanced tumor grade.

## 5. Conclusion

Somatostatin receptor 2 (SSTR2) and SHP-1 tyrosine phosphatase are two fundamental components on antiproliferative effect of somatostatin. Normal epithelial prostate cells express SHP-1 and SSTR2, but we report that the levels of expression were diminished or lost for two proteins in advanced prostate cancer with an inverse ratio between protein expression and Gleason grade of tumor. 

Further studies are necessary to determine whether the loss or up-regulation of others receptors by antiproliferative peptides may contributes to prostate tumor development and progression. Certainly, other parameters will have to be further evaluated for determinate why SHP-1 and SSTR2 expression diminish, establishing as a possible tumor suppressor gene role of this proteins in human prostate and determining the therapeutic potential of SHP-1 in clinical outcome, that is, survival and tumor recurrence.
